# Photostory—A “Stepping Stone” Approach to Community Engagement in Early Child Development

**DOI:** 10.3389/fpubh.2020.578814

**Published:** 2020-12-17

**Authors:** Abhay Gaidhane, Penny Holding, Minal Shah, Manoj Patil, Shital Telrandhe, Navnita Jadhav, Priti Kogade, Sonali Chaudhari, Quazi Syed Zahiruddin

**Affiliations:** ^1^School of Epidemiology in Public Health & Community Medicine, Jawaharlal Nehru Medical College, Datta Meghe Institute of Medical Sciences, Wardha, India; ^2^School of Epidemiology in Public Health, Datta Meghe Institute of Medical Sciences, Wardha, India; ^3^Educational Psychologist, Institute of Educational Development, Aga Khan University, Nairobi, Kenya; ^4^Jawaharlal Nehru Medical College, Datta Meghe Institute of Medical Sciences, Wardha, India; ^5^School of Epidemiology in Public Health, Jawaharlal Nehru Medical College, Datta Meghe Institute of Medical Sciences, Wardha, India; ^6^Research and Development & Community Medicine, Jawaharlal Nehru Medical College, Datta Meghe Institute of Medical Sciences, Wardha, India

**Keywords:** ECD, parenting, community engagement, rural India, photostory

## Abstract

**Background:** Capturing real-life practices through photographs provides an opportunity to create awareness and focus discussions on relevant issues in community. Photographs and narratives also engage decision makers, inspiring changes in policy and practice.

**Objective:** Paper describes development and adaptation of an integrated photostory approach documenting actions and stimulating positive change in Early Child Development (ECD).

**Methodology:** The Photostory method was built through a cyclical process to describe and explore early-childhood practices in central-India through photographs and stories. A systematic format to capture, archive and evaluate photographic material was developed. A standardized rating system was established to monitor levels of, and change in, community practices. We integrated Photostory process into routine visits undertaken during implementation of Stepping-Stones, an ECD intervention program. This paper utilized collected data to explore utility of rating framework to describe and measure behavior and to track change. We explored role of Photostory approach in engaging community in process of stimulating positive ECD experiences.

**Results:** We developed a systematic framework to support data capture, analysis, and data utilization using multistep iterative mixed method process. A total of 161 Photostories were collected (72 at baseline and 89 at endpoint). Using a rating system which measured both the structure of the tasks, and the emotional engagement of the child and parent, many activities and practices observed were evaluated as providing at least an adequate learning space. In exploring change over the implementation process, at endpoint children were more likely to be observed as more engaged in their play activities (*p* < 0.05). Parental engagement levels remained stable, toward being less actively engaged. At endpoint we observed a trend toward activities being provided for children at a level of difficulty higher than child's level of developmental. The data provided the intervention team with local examples through which to engage parents in discussions on activities that stimulate effective child exploration and learning. We were also able to demonstrate the added value of photographs in stimulating detailed discussion amongst community members on early child development.

**Conclusion:** Photostories can provide a systematic and rigorous methodology to stimulate engagement, monitor and measure change in community-based parenting interventions.

## Background

Photography is seen as an important and effective means of involving people in activities that promote health (1). Photographs provide a means to connect life experiences with scientifically based knowledge, so that people are “touched rather than indoctrinated” by health messages (2). Photographs also provide a way to express health related needs and views without the need for complex language and scientific knowledge. WHO recognizes the power of photographs to communicate—a power that transcends barriers of language, distance, and time. Photography has long been employed by the organization to inform the public of new developments in healthcare and medical technologies, to give a face to initiatives and projects that might otherwise seem unconnected, and to increase understanding of global health concerns (3).

Photo elicitation has provided an essentially qualitative research method widely used in the social and health sciences as an evidentiary tool (4). Photographs provide a visual insight which, through their description or dissemination, offer a means to assess knowledge at a specific point in time and to document the change and the development of ideas (5).

In Photostory, we have developed an innovative approach to documenting and stimulating change in social actions, initially for use in the Stepping Stones Early Childhood Development (ECD) Programme. The programme operates in two rural districts in Central India to support and promote positive and conscious caregiving practices amongst families and ECD practitioners. The Photostory technique is an adaptation of Photovoice, which uses photographs taken spontaneously, without artistic or journalistic intent, to capture personal perceptions as part of the process of stimulating social change (6, 7)^.^ The three main goals of Photovoice are to enable people to record and reflect their community's strengths and problems, promote dialogue about these issues and to engage communities directly with policymakers (6). Photostory integrates into the process of documentation and reflection a structured evaluation of the activities captured. Photographs and their accompanying narratives were used to rigorously monitor ECD practices, systematically track change, and stimulate dialogue on ECD practices.

### The Context

Stepping Stones involves a number of different approaches to stimulating change in ECD, a vital time point in development (8). The programme is designed to stimulate change in six key areas of child development and child-care: Food and Nutrition; Shelter and Care; Health and Safety; Play and learning; Psychosocial Skills; and Child Protection (9, 10). For parents of children under 3 years old, the intervention includes activities such as toy making, as well as the opportunity to participate in conversations facilitated by ECD practitioners in which information is shared on needs and practices. For children aged 3–6 years the focus is on supporting the Anganwadi (or Early Childhood) Centers to deliver a child centered curriculum.

The Stepping Stones initiative was built on the evidence that the environment and nurturing care play a vital role in shaping the interactions a child experiences (11, 12). Continuous exploration and learning in the early years of life build both positive and negative experiences, with each type of experience forming the basis of brain development and functioning, which in turn impacts upon health and well-being throughout the life course (12). The complex interactions between the parent-child and environment thus need to be understood and monitored to ensure healthy caregiving relationships essential for positive outcomes (9, 11).

In this report we describe the development of Photostory and reflect on the lessons learnt about applying an inductive learning process within a community-based intervention.

## Methodology

The Stepping Stones programme promotes positive and conscious activity by caregivers to support their children to become confident and independent learners (13, 14). The theoretical framework of the program design comes from Vygotsky's concept of the Zone of Proximal Development (ZPD). The ZPD refers to the development of independent action/skills/knowledge through the provision of adequate support and encouragement, referred to as “scaffolding” (15–17). The Stepping Stones program was delivered by 30 community volunteers trained and certified in ECD. These community volunteers were female, from the same village, able to read and write the local language (high-school education was preferred wherever available) and willing to give 2 h a day for 4 days a week (total 8 h in a week). Each community volunteer served around 8–10 beneficiaries from the same village and were supported by 1 field supervisors. The intervention included the delivery of a parenting curriculum through personalized home visits and group sessions. The intervention was delivered to 326 eligible households in 55 villages covering a population of 41,663.

Our intention in exploring the use of photographs was to enhance the monitoring and evaluation process of the programme and help us make corrections to enhance the quality and coverage of the intervention. We sought a methodology that: increases awareness of what is happening; tracks achievements and needs; and involves the community directly in that dialogue. We also sought to systematically and rigorously collect material that supports learning/training, evidence informed decisions and that can be used for advocacy with policy makers. Photostory was thus developed to provide an integrated approach to continuous learning, evaluation, and decision making. The name Photostory was used because of the intent to combine photographs with a supporting narrative. The Objective of Photostories was to capture:

- Evidence of the “learning” environment: In early childhood, every experience provides the opportunity to learn. The photostory illustrates what those experiences are and how they are structured. Each story can be explored for evidence of nurturing care, protection, and exposure to stress. They can therefore provide the evidence of whether an experience offers adequate and appropriate scaffolding. By making a comparison over time we will also explore the use of Photostory as a method to evaluate impact.
- Material to build a repository of Contextualized Resources: By illustrating example behaviors based on real life stories that are set in the local community Photostories have the potential to initiate discussion as well as trigger reflection and action. With the focus of the Stepping Stones programme on inductive learning, contextualized materials provide valuable support to the implementation of ECD interventions. We will explore the use of Photostory to stimulate engagement, and trigger conscious decision making.

### Ethical Approval

The study was first approved by the Institutional Ethics Committee Datta Meghe Institute of Medical Sciences, India and main trial was registered with Clinical trial registry of India (Trial Registry Number: CTRI/2017/05/008553) (Registered on: 15/05/2017).

Consent from the Village Head was sought to take photos in the neighborhood and community settings. The community-based volunteers took photographs on their mobile telephones/tablets as they carried out their home visiting schedules. The photos were taken unobtrusively and then consent was taken from the head of the household to use the captured materials.

### Development, Implementation, and Evaluation

**Figure 1:**
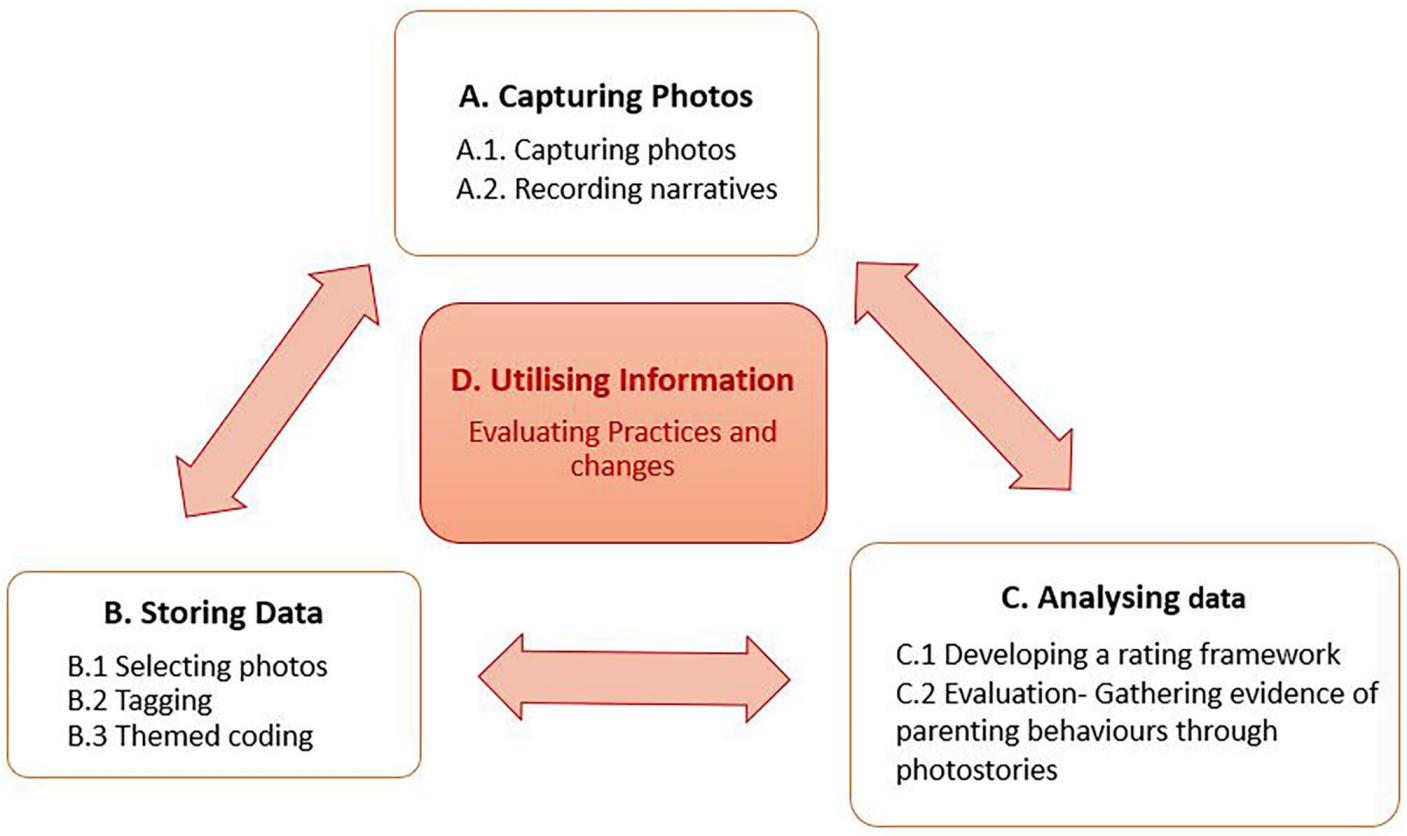
Components of Photostory.

The development of the photostory methodology was a co-creative process coordinated by a team of researchers responsible for the monitoring and evaluation of the Stepping Stones programme. We intentionally sought to develop a technique that provided information to add value to the programme. The “add” would come from the observation of real life events, while “value” would come from the triangulation of evidence from other sources using a method that was rigorous, systematic, and feasible to complete within an already complex intervention system. The different components, how to capture, store and analyse the information, developed through an iterative process outlined in Figure 1.

Following the initial design of the photostory methodology each component was evaluated as it was used, in a cycle of application and review, using feedback from the the community-based team. From this we observed the feasibility of the method proposed and identified the challenges to be addressed. Adaptations, or even re-design were then identified in collaboration with those who would actually be using the methodology, taking careful notice of what methods might cause additional challenges, and what aspects of the process engaged the users, and were seen as valuable. This was a crucial element of our design, enabling it to be culturally and contextually relevant so as to weave it into the system seamlessly. Our final step was the evaluation of information gathered through Photostory to measure behavior, track changes, make evidence-based decisions so as to stimulate further change in the Stepping Stones program. We applied a multistep iterative mixed method process to identify themes and evaluate changes in practices.

#### Capturing Data

##### Capturing Photos

Our primary challenge, while working within a limited budget, was access to and distribution of the necessary equipment, as well as the time to build skills to produce photographs with the clarity needed to support the systematic rating of the content. While other photography-based methods have used the participants themselves to collect the photographic material, these challenges precluded this as an option, at least for the initial round of data capture. Instead we used the staff delivering the intervention as the “observers,” while still directly involving parents in the process of analysis and reflection. Community based staff were trained on photo capture techniques, including composing photographs, as well as ethical issues on the collection and use of photographs. They were instructed to capture snapshots of activities they observed in the field which illustrated, in one or a series of photographs, current ECD practices in the community and homes.

##### Recording Narratives

Data collectors were trained to keep field notes, which were converted to narratives around the photographs on the day of capture. The narrative was intended to clarify the location of the activity, the proximity of the caregiver, and any other information that would increase the clarity of the story being told.

#### Storing Data

The photographs and narratives for which consent was received were handed over on the same day to central office for tagging and storage. The challenge here was to set up a system that could be applied efficiently to enter the Photostory into storage, as well as to easily identify and retrieve data as required. The system for downloading, archiving, and tagging the photos was managed by the coordination team. The keys steps identified were:

##### Selecting photos

From the photographs taken and downloaded only those photographs that were clear and in focus were archived.

##### Tagging

Each photograph was provided with a Unique Identification code that captured the date, time, and place of the story, while maintaining the confidentiality of its source. The code also linked the photograph/photographs to a transcription of its narrative.

##### Themed Coding

A data bank of photos was built up to be used as resource materials. To help in data retrieval the themes covered by the photo were identified and the photos tagged accordingly.

#### Analyzing Data

##### Developing a Rating Framework

The challenge was to identify a method that was simple enough to be applied consistently across raters while also capturing the inherent complexity of the Photostory. Other criteria were to enable comparison over time and place, and to share the findings in a clear, meaningful, and accessible format. Initial attempts to capture the complexity of each activity through an individual score failed to achieve these criteria. Instead we created an evaluation matrix, that reflected both the adequacy of the support provided, central to the ZPD concept of “scaffolding,” as well as the emotional aspects of teaching and learning that are integral to effective learning (18).

The starting point for this matrix was a description of positive parenting behaviors. A positive parent will ensure the young child is being watched over, but has the opportunity to explore, is encouraged to try new experiences, and develop old skills, and is praised for the attempt, irrespective of success or failure. The positive space will be where a balance is achieved through:

- Giving priority to a child's needs, interests, and level of development, while also drawing boundaries around safety.
- Providing clear standards of behavior and consistent monitoring, while also encouraging the child's freedom of expression.
- Including positive feedback through expressions of warmth and affection.

The Matrix was conceived as defining the “Learning Space” along 2 dimensions, that of Structure/Organization and Emotional Engagement. Within each dimension we acknowledged that a positive experience is a balance between two extremes, high vs. low, and that effective learning can be constrained by “too much” or “too little” on either of our key dimensions.

##### Dimension 1—Structure/Organization

This covers the planning of and for an event, activity, or routine, asking the questions: Are ECD opportunities being provided at all? Are they at an appropriate level for the child? Skills of Organization cover a spectrum, from disorganized; where there is no evidence of planning, no provision of materials, little or no attention to hygiene, environmental hazards; to Overly Organized, where the routines of the family or the events planned are controlled completely by the parent, allowing for no independent choice or space for the child to develop his/her own curiosities. The positive parent will anticipate and prepare to address needs, while at the same time providing the child with appropriate space to explore and play independently.

##### Dimension 2-Emotional Engagement

This covers the psychosocial aspect of participation in an event, activity, or routine. The evidence for this can be observed in the motivational support provided to the child, asking the question: Is there evidence of encouragement? Again it covers a spectrum, from no encouragement, where the child's achievements are not acknowledged, or the child is experiencing harsh discipline, to the caregiver being overly attentive, and not providing sufficient space for the child to develop his/her own choices, ideas and decision-making skills.

For each activity there are two perspectives that can be captured. One perspective is that of the Child as a Learner, and the other perspective is that of the Adult or Older child as a Caregiver, or mentor.

- From the Learner's perspective we examined **(a)** the **engagement** of the learner in the activity portrayed, and **(b)** the adequacy of the **level of independence** the activity allows. The focus is therefore on the match between the learning needs, and the learner's attention to and participation in that activity.
- From the Caregiver's perspective we examined **(a)** the **emotional engagement** of the caregiver to motivate and engage with the learner, and **(b)** the attention paid to the **organization or structure** of the task to stimulate independence.

While there is clearly some conceptual relationship between the two matrices generated, the child matrix illustrates how engaging learning activities are, while the caregiver matrix illustrates the effectiveness of the mentors (caregivers), as a measure of positive parenting, or effective scaffolding.

To be able to place each Photostory on the matrix a Rating Key was developed. Through an iterative process we sought at each step to increase the clarity of the language used, and thus show an increase in the consistency of the ratings within and between “raters.” We used a 5 point scale, with “too little” at one end of the spectrum, and “too much” at the other (rated as A to E), with the central ratings indicating a “balance,” the effective learning space. The key for rating from child and parent's perspectives are provided in Table 1.

**Table 1:** The key for rating the learning space.

**Dimensions**	**Child perspective**	**Parent perspective**
Emotional engagement	A = No engagement. B = Partial engagement. C = Active engagement. D = Signs of discomfort or irritation or anxiety. E = The child's agitation is evident.	A = No engagement. B = Partial Engagement. C = Adequate engagement. D = Signs of discomfort or irritation or anxiety. E = Parental agitation is evident.
Structure and organization	A = Task limits growth since it is very challenging. B = Learner can only partially tackle the task. C = Task well-matched. D = Task is relatively easy limiting the child to explore their potential. E = Task is too simple.	A = Parent provides too much support, dominating the activity. B = Parent's interference interrupts what the child is doing from time to time. C = Parent organizes the situation to maximize the child's independence without exposing the child to unnecessary hazard. D = Parent provides inadequate support. E = Parent leaves child without support.

##### Evaluation—Gathering Evidence of Parenting Behaviors Through Photostories

**Figure 2:**
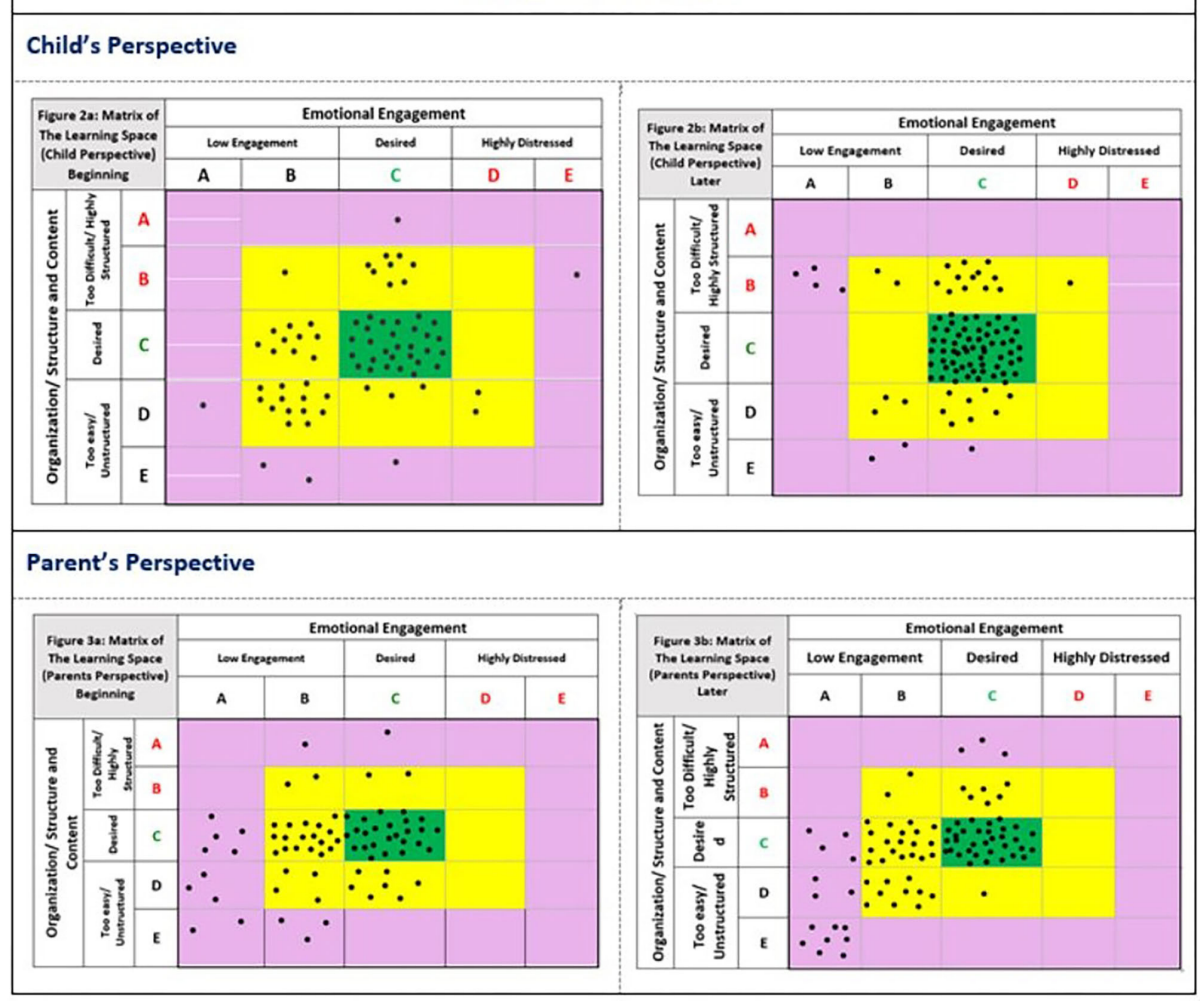
Photostories categorized using matrices of learning space from child and parent perspective at baseline and endpoint.

Data Analysis was carried out by assigning a place for each Photostory collected in the Child/Learner Matrix. If the parent or older child was present, then the Caregiver Matrix was also completed. For cross validation, two independent persons not part of the photo collection process applied the ratings. The matrix of the learning space, along with the placement of the Photostories is presented in Figure 2. It reflects the pattern of behaviors observed, and also has the potential to provide guidance on next steps, illustrated by the different colors.

- Photostories placed **in the green space** indicate effective learning. These Photostories can be used to illustrate good practice.
- A Photostory that is placed **in the yellow space** indicates that the activity, or behaviors around it, could be adjusted in some way to make the experience of learning more effective. The type of adjustment depends upon exactly where the **story is** placed (see the description of the red and gray spaces below).
- Those **in the purple space** require significant adjustments, as they were placed at the extremes of the learning dimensions. Again, the adjustments needed will depend upon which end of the dimensions of learning they fall in.
- Each dimension is graduated from A to E, highlighted by red, green, and gray colors in Figure 2.
- Those levels in red indicate a trend toward “too much or extreme,” characterized by too much interference, anxiety, over-protection, or inappropriately high levels of difficulty. For Organization/Structure this is represented by levels A or B. For Emotional Engagement by spaces D or E. When a Photostory is placed here, parents need to increase their awareness of the positive benefits of free play and exploration.
- Those levels in gray indicate a trend toward “too little,” characterized by disengagement that comes from under-stimulation, disinterest, or overly simple tasks. For Organization/Structure, these are represented by stories placed in D or E and for Emotional Engagement for those in A or B. When a Photostory is placed here, parents need to increase their awareness of the positive benefits of scaffolding matched more closely to the child's level of development.

#### Utilizing Information

##### Describing Practices and Evaluating Change

Photostories were captured by the Stepping Stones team at two time points in the programme, Baseline and Endpoint. We informed parents/caregivers about how the photographs would be stored and also about how they might be used in the future. Participants' identity was not revealed. Photographs and stories that depict a vulnerable point in the child's or family's life were carefully archived and the identity of the person was protected. We anonymized the images by blurring or pixelating faces or other identifying features. We did this with several images.

We examined the two dimensions separately, at bothBaseline and Endpoint, to explore the Learning Space, and then compared the distribution of the Photostories captured at each time point, to explore the possible change over time. The focus of the analysis was the effectiveness of the Photostory process to capture behavior and track change. The data was combined across all villages receiving the Stepping Stones intervention to quantify the Learning Space and describe local practices.

##### Results

A total of 130 photographs were captured over a period of 3 months at the baseline of the programme. Out of these, 8 photographs that were dark, blurred, or repeated were excluded from the analysis. Some Photostories had only a single photograph, while others included a series of 3–4. Thus, out of 122 photographs, a total of 72 picture-stories were analyzed, 39 photographs were of children only and 33 were of parent and child.

At endpoint, a total of 105 photographs were captured over a period of 3 months during the endpoint survey. Out of these, 16 photographs that were dark, blurred, or repeated were excluded from the analysis. Thus, out of 105 photographs, a total of 89 picture stories were analyzed, 44 photographs were of children only and 45 were of parent and child.

The demographic characteristics of the villages from which photostories were collected are described in Table 2.

**Table 2:** Demographic characteristics of participants.

**Participant/household characteristics**	**No (%)**
Mothers age in years (mean; SD)	23.79 (3.57)
**Mothers education**	
Illiterate	11 (3.37)
Primary (1–5)	12 (3.68)
Secondary (6–10)	156 (47.85)
Higher secondary	100 (30.67)
Graduate and above	47 (14.42)
Fathers age in years (mean; SD)	29.59 (3.63)
**Education group**	
Illiterate	13 (3.99)
Primary (1–5)	23 (7.06)
Secondary (6–10)	175 (53.68)
Higher secondary	82 (25.15)
Graduate and above	33 (10.12)
**Household characteristics**	
**Caste category**	
Schedule caste	24 (8.11)
Schedule tribe	141 (47.64)
Other backward classes	123 (41.56)
Open/general	8 (2.70)
**Wealth index**	
1st Quintile	49 (15.03)
2nd Quintile	58 (17.79)
3rd Quintile	83 (25.46)
4th Quintile	75 (23.01)
5th Quintile	61 (18.71)
Average family size, (mean; SD)	4.86 (1.91)
Below poverty line (as per the governments Guidelines)	145 (44.62)

We extracted evidence of the learning space provided for children and compared these across time. The results are presented below in Table 3.

**Table 3:** Proportion of activities in the effective learning space.

		**Green *N* (%)**	**Yellow *N* (%)**	**Purple *N* (%)**	***p*-value**
Child	Baseline	29 (40.3)	37 (51.4)	06 (8.3)	chi2 = 9.691 *p*-value = 0.007
	Endpoint	60 (63.8)	27 (28.7)	07 (7.4)	
Parent	Baseline	25 (34.7)	32 (44.4)	15 (20.8)	chi2 = 0.096 *p*-value = 0.952
	Endpoint	33 (37.1)	38 (42.7)	18 (20.2)	

From a child perspective, a statistically significant increase was seen in the number of photostories in the effective learning space i.e., green. From the parents' perspective photostories illustrated behaviors that more commonly fell in the yellow space, would benefit from adjustments, at both baseline and endpoint.

A statistically significant increase was found at endpoint in the number of photostories that reflect effective structure/organization of activities matching the child's learning needs (C). There was also an increase in the proportion of activities that were rated as “too difficult,” with fewer that were rated as “too easy.”

From the perspective of the parental contribution to the structure/organization of the activities portrayed, the majority of activities were at level C at both baseline and endpoint, with an increase in more difficult activities observed at endpoint (Table 4).

**Table 4:** Structure/Organization of activities from child and parent's perspective.

		**Levels of structure/organization** ***N*** **(%)**			***P*-value**
		A & B^*^	C^**^	D & E^***^	
Child	Baseline	11 (15.3)	39 (54.2)	22 (30.5)	chi2=6.035 *p*-value = 0.048
	Endpoint	20 (21.3)	60 (63.8)	14 (14.9)	
Parent	Baseline	6 (8.3)	47 (65.3)	19 (26.4)	chi2 = 0.689 *p*-value = 0.708
	Endpoint	11 (12.4)	56 (62.9)	22 (24.7)	

* *A & B-Highly structured or highly organized*.

** *C-Parent organizes the situation to maximize the child's independence without exposing the child to unnecessary danger*.

*** *D & E-Unstructured or too easy*.

Again, at endpoint a statistically significant increase was observed in the number of photostories portraying children effectively engaged in activities. Parental engagement levels remained stable, with no examples observed of high levels of interference (Table 5).

**Table 5:** Emotional engagement of activities from child and parent's perspective.

		**Levels of emotional engagement** ***N*** **(%)**			***P*-value**
		A & B^*^	C^**^	D & E^***^	
Child	Baseline	27 (37.5)	42 (58.3)	3 (4.2)	Chi2 = 18.041 *p*-value = 0.000
	Endpoint	11 (11.7)	82 (87.2)	1 (1.1)	
Parent	Baseline	37 (51.4)	35 (48.6)	0 (0)	Chi2 = 0.001 *p*-value = 0.970
	Endpoint	46 (51.7)	43 (48.3)	0 (0)	

* *A & B-Low engagement*.

** *C-Fully engaged/signs of parental interest*.

*** *D & E-High distressed*.

In addition to a more quantitative use of the data for impact analysis we also explored the different ways in which the data could be summarized, interpreted, and shared.

To explore a method that makes the data accessible to the community we also examined the pattern of photostory placements using a visuo-spatial display of the data (Figure 2). The patterns described above can be more clearly seen in this format. The green area indicates the effective learning space, the yellow area, adequate learning, that could be better matched to children's learning needs, and the purple area indicates the activities that require significant adjustments for effective learning.

Figure 2 illustrates that even at baseline the concentration of activities is toward the center, the positive learning space. The most noticeable shift for children from baseline to endpoint was toward observing a higher level of engagement in the tasks. We can also clearly see that there were no examples captured of Photostories illustrating high stress activities. At endpoint, however, there are examples of activities rated as being poorly matched to the child's developmental levels, suggesting a trend toward tasks becoming more difficult.

Amongst parents, the most noticeable pattern overall was the concentration of behaviors from positive ratings at the center, down toward the bottom left-hand side, denoting low involvement and low engagement. There is also a, smaller, group of behaviors which suggest activities that are too difficult or too highly structured. There is an absence of activities toward the right, associated with more intrusive parenting styles.

Displaying the data in this way draws attention to the the need for parents to better guide their children toward activities more closely matched to their developmental level.

### Understanding Local Practices

The details of the stories themselves provides greater insight into the meaning of the patterns described through the ratings of learning Space.

At the Formative stage, that is at baseline, Photostories reflect the positive practices already existing in the villages. This was evident both from the scope and range of activities observed. While the play environment was often observed to be appropriate for optimal growth and development, and the children were in safe spaces, only a few examples of conscious and careful practices in hygiene were found. In addition, the play activities seldom demonstrated actual parental involvement in the child activities. The children were seen to play by themselves, while the parents remained busy with their domestic chores, giving limited direction or encouragement.

We examined closely at the exact nature of the activities captured, comparing the baseline and endpoint stories on a village by village basis. The following stories illustrate the shift from the periphery of the matrix (purple and yellow) at baseline toward the center at the endpoint (green zone) taken in the same village.

#### Village 1

Baseline: “*Two children are seen sitting outside the house waiting for their parents. The younger boy is not wearing clothes. The children look tired. The door is locked. They have a school bag with them, perhaps they have returned from the school* (**Photo-Story No. 2 Base-Line Archives**).”

Endpoint: “*The mother of a child has to work in the farm and as she could not leave the child unattended, so she kept her child with her sister in law and there was a neighbour*'*s kid too in the house. The children were happy and playing together* (**Photo-story No. 1 Endpoint Archives**).”

#### Village 2

Baseline: “*A child standing in the vicinity of the house. The child is barefoot. A woman is filling water in the utensils from the tap. Another woman is sitting outside the house. A man is sleeping on a cot nearby. No interaction was evident with the child*. (**Photo-Story No. 2 Base-Line Archives**).”

Endpoint: “*A child was seen imitating her grandmother sweeping the floor. The grandmother was very much engaging with the child and she took the child away from the goats present nearby, while doing her work*. (**Photo-story No. 27 Endpoint Archives**).”

#### Village 3

Baseline: “*The child is playing with picture book, alone. Mother is doing her household chores. Grandma is talking with another lady passing by the road. Father is putting fodder to the cows in the courtyard. The child was playing unguided and exploring his environment*. (**Photo-story No. 28 Baseline Archives**).”

Endpoint: “*Mother and baby are looking at the chart pasted on the wall. Child wants to explore his environment. The baby is pointing to the pictures on the chart. As the baby is pointing, Mother is interacting and was very much engaging with the child and praising him. perhaps she is teaching her child – This is a ball. It is red in color*. (**Photo-story No. 87 Endpoint Archives**).”

#### Village 4

Baseline: “*The wooden plank is placed on the open drainage to take the motorcycle across, but the two children were sliding over that wooden plank, as a slide. They are sliding one after another. While playing this game, they seem to be quarreling with each other about who will go first and not waiting for their turn. A grandfather sitting on the porch. Both the children were unattended*. (**Photo-story No. 13 Baseline Archives**).”

Endpoint: “*Two girls and one boy are playing a popular outdoor hopping game, also called ‘tikker billa’. A grid is drawn on the ground and numbered. Players are taking turns and throwing an object, usually a small flat object over the numbered blocks. They are hopping on one/two legs across blocks to pick up the object to finish the turn. They are very much into the game. A mother and neighbor lady are sitting roadside and enjoying their child's game and cheering them for successful completion of their turns*. (**Photo-story No. 6 Endpoint Archives**).”

### Community Engagement

We later shared photographs with the community, with residents both from the intervention arm, and from the control arm of the main study. We organized the group meeting of caregivers in all intervention villages. Around 8–10 caregivers participated in the group meetings. Photographs were shown in groups and participants were asked to express their views on the activity shown.

We observed that photographs provoked detailed analysis of the structure of the activities, as well as of the levels of engagement of the “actors” illustrated in the photographs. This technique readily triggered reflections of their personal world and inner thoughts. The participants discussed various emotions like fun, joy, fear seen in the pictures, and many of them shared their own life experiences related to these emotions.

The parents and grandparents also offered insights of parenting, embedded in their own experiences of childhood. Visualizing themselves in the story helped participants to identify the areas they can strengthen or to improve in their own parenting practices. This exercise also encouraged the participants to acknowledge the positive practices in which they are already engaged, as well the significant role they can play in the child's development. At the end of each session participants were asked for their key take away. Examples of these reflections are:

*A Grandfather observed, “I am going to take my grandson for a ride on my bicycle and tell my friends the fun they are missing*.

*A mother expressed, “While feeding, taking care, and dressing the baby, I will talk to the baby, and will tell him how much I love him. I will interact with him. I have not given enough time to him, but now I will surely do by best to engage, while doing the household chores.”* *A father pledged, “From now on I will play with my daughter every day. We will build a cardboard doll house together and put doors and windows in that house. I will praise her while making doll house. My daughter will be very happy.”*

## Discussion

### Developing the Technique

In comparison to Photovoice and Photo elicitation, our Photostories approach provided information that can be more directly linked to social action plans as it describes both the level and direction of need (2). We were able to develop a systematic, structured, and feasible process to explore the practices and behavior of rural communities from central India. This analysis was based around two key dimensions of Early Child Development (ECD), Emotional Engagement and Structural Organization. The technique provided an engaging and robust methodology through which to strengthen awareness and understanding of ECD, adapt interventions to meet specific and relevant needs, and generate an evidenced based dialogue on progress and change.

### Evaluating Practices and Change

The approach helped us understand community ECD activities/behaviors in relation to effective learning by exploring in detail real-life events. The rating framework guided the analysis to reveal the type and form of activities that were already available in the community, while also providing a systematic approach to monitoring change. There was a noticeable shift of activities toward more effective learning from the child's perspective, most particularly in the greater engagement observed in the children over time. However, the activities from the parental behaviour's perspective showed a very minimal shift, possibly with a trend toward exposure of children to more difficult tasks. At a program level this information is useful to direct next steps. It suggests that future conversations with families will need to address in more detail the concept of “scaffolding.” This would involve guiding parents to closely observe their children's skills and to work together with parents to build up a bank of activities that are more closely matched to the developmental level of their child or children. Photostories will be able to provide both a focus to that conversation, as well as method through which change can be recorded and analyzed.

Through the details of the Photostories, we noted shifts in the interaction between parents and their children which the numeric data represents less effectively. We observed, for example, a change from baseline to endpoint in the way in which parents engaged with their children while carrying out daily chores. At baseline, the photostory would describe young children sitting alongside a busy parent. At endpoint, the photostories showed parents involving their children directly in the activity, increasing both the engagement of the child, and the level of communication between parent and child. This level of detail, embedded as it is in the local setting, provides data that is accessible and meaningful to the community.

Using photostories also provided a way to monitor the learning of the service delivery team. We observed that participating in photo capture and analysis stimulated a more detailed understanding of the daily experiences of the community. The evidence of positive behaviors already existing in the community provided the foundation for the field team to develop a more collaborative/interactive approach to conversations with families. They described Photostories as a “fun exercise,” that also provided a valuable self-reflective tool. It provided the field team with useful information to guide their activities and identify areas of work that need more attention. In addition to taking the photographs, the discipline of writing the accompanying narratives was found to help better understand the specific needs of the community, and of individual families. The field team were observed to use the photostories to inform the adaptation of their materials to match to the needs and lives of those they were working with.

### Building a Repository of Materials to Support Behavior Change

Awareness or conscious caregiving emerges from reflection on what is happening in both the learner (child) and the mentor or guide (caregiver). The hypothesis is that greater awareness of what is already happening will ensure that activities will be more appropriately matched to children's learning needs, and that this matching will become a greater part of caregiving behaviors. We were able to successfully use this technique to raise awareness of ECD practices in the community. The field team found that using local examples made the process of illustrating the concept of positive parenting more straightforward. They were also able to draw the families into the conversation in a more interactive way, through affirming the skills and abilities that already exist. This enabled subsequent dialogue to build upon a positive foundation.

Photostory was also effective in generating interest on the part of the community to take part in the study. Unlike interviews, surveys, and even other form of discussion groups, which often fail to catch the attention of the community, parents readily shared their own opinions and ideas when the session began with looking at photographs. The technique provided a tool for parents to reflect on and use their own experiences growing up to make conscious decisions about what practices they should promote intentionally and mindfully with their own children.

### Strengths, Limitations, and Mitigation of Risk Associated With Study

The main strength of the study was the demonstration of the adaptability of the process to the local context and of the ease with which participants could be directly involved in the evolution of the intervention. The process developed is easily replicated across different settings, as the content is, by definition, context specific.

We were also able to develop a rigorous rating scale, which links parent and child behaviors to guidance on promoting positive parenting practices. The rating framework was both systematic and informative, identified variability within the population and recorded change at both the individual and community level.

While we were able to demonstrate that within our programme the use of the Photostories technique contributed positively to team training and to community engagement we feel that we are only beginning to exploit the technique as a means of programme evaluation. For the Stepping Stones program the conclusions drawn on the impact of the intervention that this data provides is only preliminary.

Ethical considerations are a challenge. Issues of safety, respectful representation of participants in reports, and how images are used are all important considerations, and may influence the selection of stories to capture or share (1, 2). The validity of the rating scale will be enhanced by creating a more systematic framework of data capture, to reduce the potential bias in the selection of the initial shots around which the stories are based. We also need to explore the number of photostories required to provide an adequate sample.

In future steps we intend to bring families more directly into data capture, to explore the ability of this technique to both directly measure and enhance the shift from awareness to practice within the home. In addition, the framework itself needs to be validated through the exploration of the relationship between the concepts captured and child development outcomes.

## Conclusion

The Photostory approach revealed that many of the activities in which young children are involved fall within an adequate learning space, highlighting the level of competency already existing in the community. This awareness heightens the sensitivity of the intervention team to their role as a facilitator, rather than that of a trainer, further supporting the value of an inductive rather than directive approach to implementation. Reflecting on the details of stories also increased our awareness of which aspects of the learning environment could be improved to move the narrative from “being effective” to “being highly effective.”

As a next step we intend to include the community in the processes of monitoring change and evaluate how Photostory contributes to building positive self-reflective practice.

## Data Availability Statement

The raw data supporting the conclusions of this article will be made available by the authors, without undue reservation.

## Ethics Statement

The studies involving human participants were reviewed and approved by Institutional Ethical Committee, Datta Meghe Institute of Medical Sciences (DU). Written informed consent to participate in this study was provided by the participants' legal guardian/next of kin.

## Author Contributions

AG led the design as primary author, analyzed data, and led the write-up. PH closely helped in the design, the resolved discrepancy, aided in analysis and write-up. MS closely helped in the design, and assisted with the write-up. MP, ST, and QZ closely helped in the design, provided statistical inputs for analysis, and assisted with the write-up. NJ, PK, and SC assisted with the write-up. All authors contributed to the article and approved the submitted version.

## Conflict of Interest

The authors declare that the research was conducted in the absence of any commercial or financial relationships that could be construed as a potential conflict of interest.
